# Assessing malnutrition in patients with nasopharyngeal carcinoma: Diagnostic protocol for the development and validation of a new nutritional assessment tool

**DOI:** 10.1371/journal.pone.0300067

**Published:** 2024-03-25

**Authors:** Pengpeng Wang, Kim Lam Soh, Salimah Binti Japar, Huzwah binti Khazaai, Jinlian Liao, Yanping Ying, Chuanyi Ning, Li Xue, Xiao Pan

**Affiliations:** 1 Nursing College of Guangxi Medical University, Nanning, Guangxi, China; 2 Department of Nursing, Universiti Putra Malaysia, Selangor, Serdang, Malaysia; 3 Department of Biomedical Sciences, Universiti Putra Malaysia, Selangor, Serdang, Malaysia; 4 Department of Nursing, The First Affiliated Hospital of Guangxi Medical University, Nanning, Guangxi, China; 5 Department of Nursing, The Second Affiliated Hospital of Guangxi Medical University, Nanning, Guangxi, China; Longgang Otorhinolaryngology Hospital & Shenzhen Key Laboratory of Otorhinolaryngology, Shenzhen Institute of Otorhinolaryngology, CHINA

## Abstract

**Introduction:**

There is currently no gold standard or specific nutritional assessment tool to assess malnutrition in patients with nasopharyngeal carcinoma (NPC). Our study aims to develop a new nutritional assessment tool for NPC patients.

**Methods and analysis:**

NPC patients will be required to complete a risk factor questionnaire after obtaining their informed consent. The risk factor questionnaire will be used to collect potential risk factors for malnutrition. Univariate and multivariate logistic regression analyses will be used to identify risk factors for malnutrition. A new nutritional assessment tool will be developed based on risk factors. The new tool’s performance will be assessed by calibration and discrimination. The bootstrapping will be used for internal validation of the new tool. In addition, external validation will be performed by recruiting NPC patients from another hospital.

**Discussion:**

If the new tool is validated to be effective, it will potentially save medical staff time in assessing malnutrition and improve their work efficiency. Additionally, it may reduce the incidence of malnutrition and its adverse consequences.

**Strengths and limitations of this study:**

The study will comprehensively analyze demographic data, disease status, physical examination, and blood sampling to identify risk factors for malnutrition. Furthermore, the new tool will be systematically evaluated, and validated to determine their effectiveness. However, the restricted geographical range may limit the generalizability of the results to other ethnicities. Additionally, the study does not analyze subjective indicators such as psychology.

**Ethics and dissemination:**

The ethical approval was granted by the Ethical Committee of the First Affiliated Hospital of Guangxi Medical University (NO. 2022-KT-GUI WEI-005) and the Second Affiliated Hospital of Guangxi Medical University (NO. 2022-KY-0752).

**Clinical trial registration number:**

ChiCTR2300071550.

## 1 Introduction

Nasopharyngeal carcinoma (NPC) is a prevalent head and neck malignancy characterized by high invasiveness and early metastasis. Patients with NPC are often diagnosed at an advanced stage due to the lack of early symptoms [1]. While relatively rare in comparison to other types of cancer, there were 133,354 new cases of NPC in 2020 [2]. However, the distribution of NPC cases is highly geographically skewed, with over 70% of new cases occurring in East and Southeast Asia [3, 4]. A recent study has shown that in 2020, the provinces of Hainan, Guangxi, and Guangdong in southern China had the highest rates of years of life lost due to NPC among all provinces in China [5]. Furthermore, the proportion of NPC-related deaths among all cancer deaths in China remained at approximately 1.0% from 2005 to 2020. However, the overall number of NPC-related deaths increased by 7.3% over the same period [5].

The prevalence of malnutrition in cancer patients worldwide is reported to range from 20% to over 70% [6]. The deleterious effects of malnutrition in cancer patients have been well documented, including weight and muscle loss, compromised immune function, increased susceptibility to infections, psychosocial stress, diminished quality of life, and treatment toxicity [6]. According to recent research, the occurrence of malnutrition among NPC patients varies from 5.3% to 94.8% [7–9]. Malnutrition in NPC patients may compromise the immune system, prolong hospitalization, and diminish the effectiveness of treatment [10–12]. Moreover, several studies have suggested that there is a correlation between malnutrition and poor survival outcomes in NPC patients [13].

Prevention of malnutrition is feasible through early identification of at-risk patients. To comprehensively manage malnutrition in hospitals, all patients should be systematically screened for nutritional risk on admission, and a detailed assessment of the nutritional status of those identified as being at high risk should be carried out [14]. Subsequently, malnourished or high-risk patients should receive individualized nutritional interventions that are suitable for their specific needs [15]. Although the importance of this process is recognized, it is not consistently implemented. A “NutriDay” survey comprising 21,000 patients across 325 hospitals in 25 European countries showed that established detection routines for malnutrition were only implemented in 52% of hospitals (with a range of 21% to 73%) [16]. Although malnutrition is common and nutritional assessment is critical, there is currently no universally accepted standard for nutritional assessment [17, 18]. However, commonly used malnutrition assessment tools have limitations [19]. For example, PG-SGA is complex and time-consuming, and its results also depend on the experience of the observer [37]. While GLIM remains an expert consensus, and its clinical effect still needs to be verified [38]. The applicability of MNA to Asians may be limited due to the absence of race-specific metrics, and the definition of BMI in MNA may need to be adapted to account for racial differences [19]. To address these gaps, the study aims to develop a specific tool for assessing malnutrition in NPC patients. The new tool has significant advantages. It not only has easily obtainable assessment indicators, saving time, but also includes evaluation indicators specific to the nutritional risk factors of NPC patients. The tool may provide a more personalized and accurate assessment for NPC patients.

## 2 Methods and analysis

The primary aim is to develop and validate a new nutritional assessment tool for NPC patients that is based on risk factors for malnutrition. The research timeline spans from June 2023 to April 2024 and will be conducted through a two-phase approach. We have access to information that could identify individual participants during or after data collection.

### 2.1 The Phase I

#### 2.1.1 Objectives

The aim of Phase I is to develop, evaluate, and internally validate a new nutritional assessment tool for NPC patients.

#### 2.1.2 Study design

The study is designed as a prospective, cross-sectional diagnostic study.

#### 2.1.3 Study settings and participants

The study will be conducted at the First Affiliated Hospital of Guangxi Medical University, Nanning, Guangxi, China, from June 2023 to December 2023.

Eligible participants for the study will be recruited in June 2023 based on predefined inclusion and exclusion criteria.

(1) Inclusion criteria
  (i) Patients with newly diagnosed NPC confirmed by pathology; (ii) Patients aged more than 18 years; (iii) Patients who have no history of other malignancies than cancer.
(2) Exclusion criteria
  Patients who have life-threatening diseases, mental disorders, or intellectual disabilities will be excluded.

#### 2.1.4 Study tools

(1) The risk factor questionnaire
  The risk factor questionnaire includes the following potential risk factors: socio-demographic data (gender, BMI, and age), lifestyle habits (smoking and drinking history), treatments (the number of chemotherapy cycles and radiation treatments), disease state (acute or chronic inflammation, tumor stage), anthropometry (measured by calf circumference), and blood indices (hemoglobin, total protein, albumin, pre-albumin, and retinol-binding protein).
(2) The Patient-Generated Subjective Global Assessment (PG-SGA)
  The PG-SGA is a comprehensive tool designed to assess malnutrition in cancer patients [20]. The PG-SGA evaluates seven aspects, which include body weight, food intake, symptoms, activity and physical function, disease and nutritional needs, metabolic needs, and physical examination [20]. The patient evaluates the first four aspects, while medical staff assess the last three. The PG-SGA score ranges from 0 to 1 for no malnutrition, 2 to 3 for suspicious or mild malnutrition, 4 to 8 for moderate malnutrition, and ≥9 for severe malnutrition [20].

#### 2.1.5 Sample size

The sample size calculation was based on previous research conducted by Riley et al. [21]. We included 15 predictor variables in the risk factor questionnaire. Based on the findings reported by Song et al. [22], a PG-SGA score of 2 or higher was utilized as a diagnostic criterion for malnutrition. The incidences of malnutrition and well-nourished in NPC patients were 0.599 and 0.401, respectively [22]. For logistic regression models with outcome proportions of 0.4, the corresponding max(R^2^_cs_) value is 0.74 [21]. For binary outcome, R^2^_cs_ = 0.5* max(R^2^_cs_) = 0.37. The sample size was calculated using the R language (version 4.2.1) with the pmsampsize package. The programming to calculate sample size was “(type = “b”, rsquared = 0.37, parameters = 15, prevalence = 0.401)”. After comparing the calculated sample sizes, the largest sample size was 370.

#### 2.1.6 Data collection

We will use the risk factor questionnaire and PG-SGA to collect data. Data will be collected through face-to-face interviews, clinical examinations, and anthropometric measurements. To ensure data quality, the investigator responsible for data collection will have their work checked by another investigator, with any discrepancies to be resolved through a discussion between the investigators. The data collection process will continue until the targeted sample size has been attained.

#### 2.1.7 Data analysis

The analysis of the data will be performed using SPSS 26.0 and R software 4.2.1. Quantitative data will be presented using medians with interquartile ranges [P25, P75] and means ± standard deviations. For univariable analysis, we employed the t-test, chi-squared, and Wilcoxon rank-sum tests for continuous, categorical, and graded or skewed distribution variables, respectively. Variables with p-values less than 0.1 will be selected for the multiple logistic regression model, with variables having a p-value less than 0.05 considered statistically significant. The nomogram and calibration plot will be generated using the “rms” package of R software 4.2.1. The receiver operating characteristic (ROC) curves will be plotted, and the area under the ROC curve (AUC) values will be calculated using the “pROC” package of R software 4.2.1. Double-checking of data entry will be implemented to ensure consistency.

#### 2.1.8 Study procedure

1. Three hundred and seventy NPC patients will be recruited at the First Affiliated Hospital of Guangxi Medical University.
2. NPC patients will be divided into well-nourished and malnourished groups according to the PG-SGA. The risk factor questionnaire will be used to collect potential risk factors for malnutrition in NPC patients. These potential risk factors will be analyzed using univariate and multivariate logistic regression analyses to identify risk factors for malnutrition in NPC patients.
3. Based on the identified risk factors, a set of mathematical equations for a new nutritional assessment tool will be developed. The tool will be graphically represented as a nomogram. Our aim is to provide a visual and intuitive method that enables healthcare professionals to quickly assess an individual’s risk of malnutrition based on specific risk factors.
4. The new tool’s performance will be evaluated using measures of discrimination and calibration. Specifically, discrimination will be assessed by the ROC curve and AUC value, while calibration will be evaluated using a calibration plot.
5. The internal validation of the new tool will involve bootstrapping with 200 replications. ROC curves will be generated, and AUC values will be calculated.

### 2.2 The Phase II

#### 2.2.1 Objectives

The aim of Phase II is to externally validate the new nutritional assessment tool.

#### 2.2.2 Study design

The study is designed as a prospective, cross-sectional diagnostic study.

#### 2.2.3 Study settings and participants

The study will be conducted at the Second Affiliated Hospital of Guangxi Medical University, Nanning, Guangxi, China, from January 2024 to April 2024.

Eligible participants for the study will be recruited in January 2024 based on predefined inclusion and exclusion criteria. The inclusion and exclusion criteria for participant eligibility in this phase are the same as in Phase I.

#### 2.2.4 Study tools

The new tool and PG-SGA will be the study tools in Phase II.

#### 2.2.5 Sample size

Currently, there are no generally accepted methods for calculating the sample size required for external validation of risk prediction models. However, prior investigations [23, 24] have put forth a recommendation that external validation studies should incorporate no fewer than 100 events and 100 nonevents to ensure accurate and precise estimates of performance measures. Thus, to comply with this recommendation, at least 100 well-nourished NPC patients and 100 malnourished NPC patients will be recruited to validate the new tool in Phase II.

#### 2.2.6 Data collection and analysis

The new tool and PG-SGA will be used to collect data to assess malnutrition in NPC patients. The external validation will be performed using the ROC curve, AUC value, and calibration plot. The ROC curve will be performed, and the AUC value will be calculated using MedCalc software. The calibration plot will be generated using the “rms” package of R software 4.2.1.

#### 2.2.7 Study procedure

1. At the Second Affiliated Hospital of Guangxi Medical University, 100 well-nourished and 100 malnourished NPC patients will be recruited, using PG-SGA as the standard for malnutrition assessment.
2. The new tool will be utilized to assess the nutritional status of these 200 NPC patients.
3. The assessment results obtained with the new tool will be analyzed against the PG-SGA to calculate AUC for evaluating NPC patients’ nutritional status. Additionally, ROC curves and calibration plots will be generated to visually demonstrate the accuracy and reliability of the new tool.

### 2.3 Study flow chart

**Fig 1:**
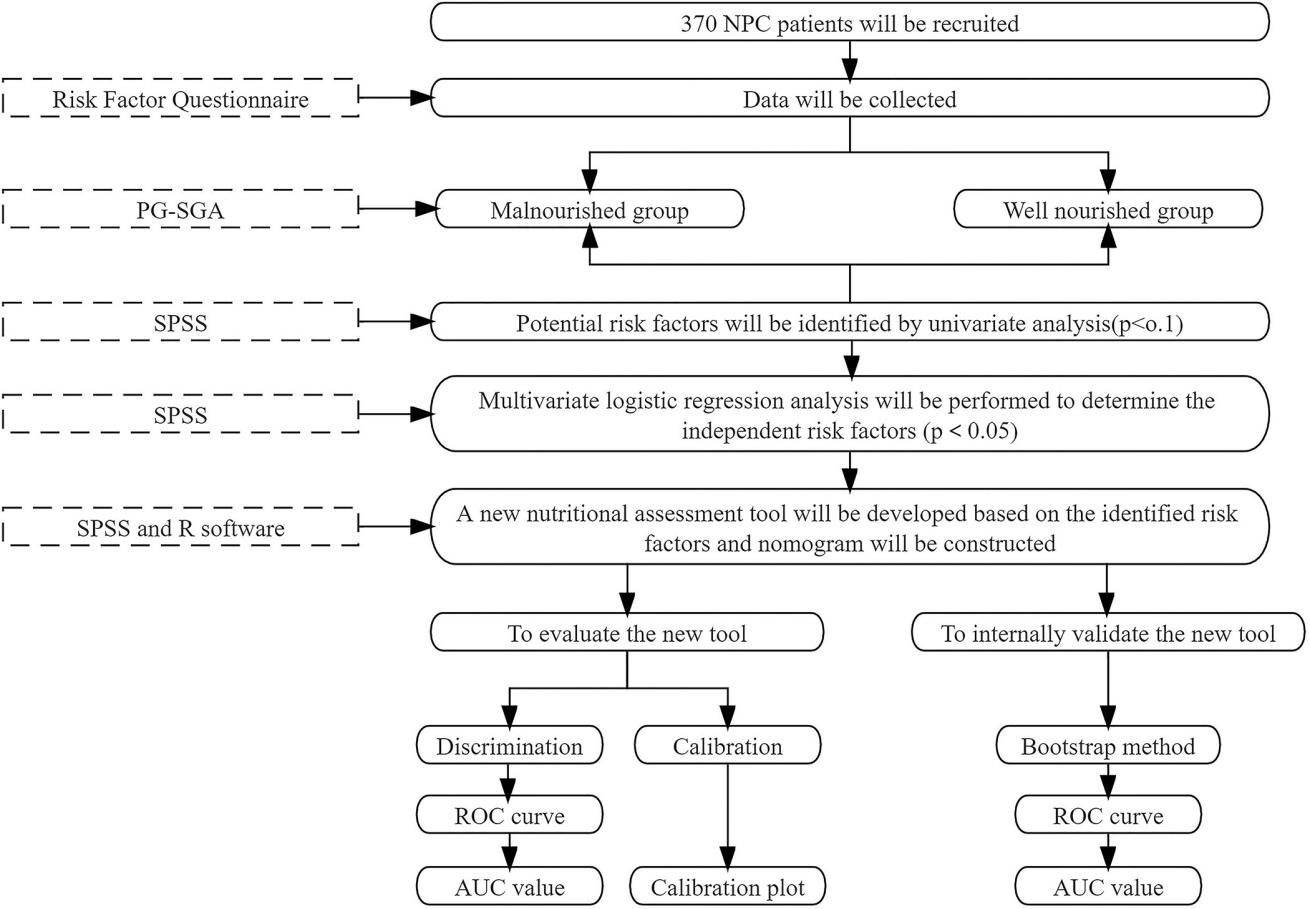
The flow chart of Phase I.

2.3.1 The flow chart of Phase I is shown in Fig 1.

**Fig 2:**
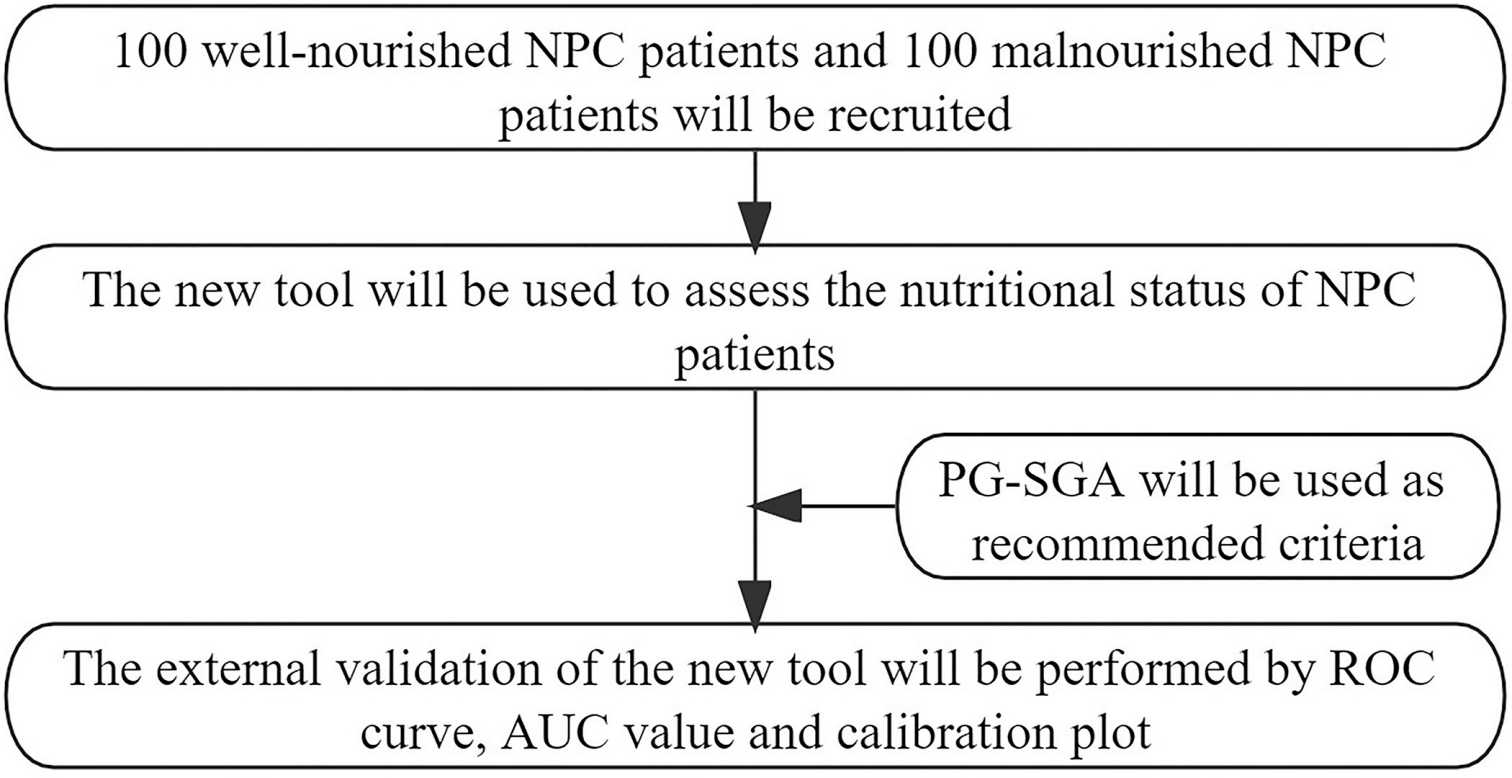
The flow chart of Phases II.

2.3.2 The flow chart of Phases II is shown in Fig 2.

### 2.4 Ethics and dissemination

The proposed study will conform to the international guidelines of Good Epidemiology Practice and the Declaration of Helsinki. Ethical approval was obtained from the Ethical Committee of the First Affiliated Hospital of Guangxi Medical University (NO. 2022-KT-GUIWEI-005) and the Second Affiliated Hospital of Guangxi Medical University (NO. 2022-KY-0752). Furthermore, prior to data collection, written consent will be obtained from all NPC patients.

### 2.5 Quality control

To ensure the rigor of the study, several methods and strategies have been implemented. Firstly, a rigorous design process was followed for the risk factor questionnaire, which involved extensive solicitation of expert opinions and suggestions as well as a thorough literature search. The initial questionnaire was reviewed and revised by experts, and pre-testing was conducted on 10% of the sample size among NPC patients. The feedback collected was used to revise the questionnaire.

Furthermore, two investigators will undergo unified training, and they will be responsible for collecting and managing the data from NPC patients. To prevent the occurrence of duplicate records, the data was matched independently with the unique ID numbers of participants.

After each patient completes the questionnaire, the interviewer will check on-site, and the participant will be asked again to complete the questionnaire if there are any omissions. All data entry will be double-checked and double-entered.

### 2.6 Patient and public involvement statement

We involved NPC patients in the development of the risk factor questionnaire. We sought feedback from patients on the questionnaire’s clarity, ease of understanding, and acceptability. During the study’s implementation phase, we will work closely with NPC patients to ensure that they understand the study’s purpose and procedures. Finally, we will seek patients’ feedback on the new nutritional assessment tool’s usefulness and ease of use.

### 2.7 Preregistration

The study was pre-registered in the Chinese Clinical Trial Registry (Registration No.: ChiCTR2300071550)

## 3 Discussion

Malnutrition is very common among NPC patients, and it can lead to adverse outcomes [25–27]. Pan’s survey of 102 NPC patients found that 5.3% (5/95), 19.6% (18/92), and 94.8% (36/38) of patients had NRS2002 scores <3 and PG-SGA scores ≥4 before induction chemotherapy, before radiotherapy, and at the end of radiotherapy, respectively [7]. Similarly, Wan surveyed 113 NPC patients and found that 16.8% of patients were malnourished before radiotherapy, while 91.2% of patients were malnourished at the end of radiotherapy according to the GLIM criteria [9]. Additionally, a survey conducted in Kenya discovered that 35% of NPC patients experienced significant weight loss [28]. Furthermore, another research group has found that 53.1% of the NPC patients were malnourished according to PG-SGA [8]. Song surveyed 2819 NPC patients from multiple cities in China and found that patients with PG-SGA score ≥2 and PG-SGA score ≥4 accounted for 59.9% and 37.4%, respectively [22]. Malnutrition could weaken the immune system, prolong hospital stays, and affect treatment efficacy [10–12]. Moreover, several studies have shown that malnutrition is linked to poorer survival outcomes in NPC patients [13, 29].

There are different studies using different malnutrition assessment tools to identify risk factors for malnutrition in NPC patients. Jin conducted a retrospective study on 117 patients with stage III-IVa NPC and found that those who experienced grade ≥ 3 radiation-induced oral mucositis during radiotherapy had worsened malnutrition, and pre-albumin level was identified as a predictive marker for weight loss in this patient population [30]. In another study, Hong found that moderate nutritional status before radiotherapy, high side effect score, depression, the late stage of the disease, female gender, and old age were the risk factors for poor nutritional status in NPC patients [31]. Similarly, Irungu discovered that stage IV NPC was a predictive marker for weight loss and low serum albumin levels [28]. Chen’s research revealed that NPC patients with higher symptom severity, more severe trismus, higher levels of depression, completion of treatments within 1 year, advanced stage, and female gender were more likely to be at risk of malnutrition or have malnutrition [32]. Qiu discovered that concurrent chemotherapy, Karnofsky performance status, radiation techniques, insomnia, N stage, body mass index, and global quality of life were independent prognostic factors for significant weight loss in NPC patients [33]. Finally, Xueyan utilized PG-SGA to assess malnutrition and identified economic status, overall quality of life, oral mucositis, anemia, prealbumin, weight loss during chemoradiotherapy, and clinical stages as factors for malnutrition in NPC patients [34].

Compared to previous studies, PG-SGA will be used to identify the risk factors for nutritional risk in NPC patients. The PG-SGA has been recommended by the Oncology Nutrition Dietetic Practice Group of the Academy of Nutrition and Dietetics as a comprehensive nutrition assessment tool for cancer patients [35]. Moreover, this study will comprehensively analyze the demographic data, disease status, physical examination, and blood sampling. Finally, our study had a large sample size. The identification of risk factors will provide a theoretical basis for nutritional screening, malnutrition prevention, assessment, and nutritional support in NPC patients. Identifying these risk factors would be beneficial for clinicians and healthcare practitioners in the management of NPC patients.

While malnutrition is a common problem, there is no universally accepted gold standard for the assessment of malnutrition [36]. Interobserver variability, difficulty in reproducibility, technician experience, time-consuming nature of some tools, expense of others, lack of validation for some tools, etc. may be the main reasons for the lack of a gold standard for malnutrition assessment. Additionally, the heterogeneity of the population being assessed as well as the setting in which malnutrition is assessed can also affect the definition of a gold standard.

The PG-SGA, Minimal Nutrition Assessment (MNA), and Global Leadership Initiative on Malnutrition (GLIM) are commonly used nutritional assessment tools. However, commonly used malnutrition assessment tools have many shortcomings. PG-SGA is generally considered the gold standard for assessing malnutrition in cancer patients, but it is complex and time-consuming, and its results also depend on the experience of the observer [37]. Although GLIM has unified the diagnostic criteria for malnutrition to a certain extent, it remains an expert consensus, and its clinical effect still needs to be verified [38]. The applicability of MNA to Asians may be limited due to the absence of race-specific metrics, and the definition of BMI in MNA may need to be adapted to account for racial differences [19].

This study will develop the first malnutrition assessment tool for NPC patients based on risk factors for malnutrition. This new tool will be proven to be an effective tool and useful for clinical applications, which will help to unify the standard of malnutrition assessment for NPC patients, assess malnutrition early, give nutritional treatment and support in time, improve work efficiency, and reduce adverse outcomes caused by malnutrition.

## 4 Limitation of study

Compared to other malignancies, NPC is a rare neoplasm exhibiting marked ethnic predisposition and regional variations, with a high incidence in densely populated regions of South China and Southeast Asia. The potential limitations of this investigation include a restricted geographical range and a limited sample size, as the study will be conducted solely in China. Subsequent studies should extend beyond China, ensuring the validation of the study’s findings. Furthermore, the exclusive use of an Asian population in this study limits the generalizability of our results to other ethnicities.

This study only comprehensively analyzes objective factors such as socio-demographic data, lifestyle habits, treatments, disease state, anthropometry, and blood indices, but does not analyze subjective indicators such as psychology.

## 5 Conclusions

This study will help to clarify the risk factors for malnutrition in NPC patients, provide specific nutritional assessment tools for NPC patients, improve the work efficiency of hospital staff, and help medical staff to assess malnutrition earlier and faster and provide timely nutritional support and treatment to reduce the incidence of malnutrition in NPC patients.

## Supporting information

S1 File Inclusivity in global research. (DOCX)
