# Supplementary material for: Assessing malnutrition in patients with nasopharyngeal carcinoma: Diagnostic protocol for the development and validation of a new nutritional assessment tool
Source: PLoS One. 2024 Mar 25;19(3):e0300067. doi: 10.1371/journal.pone.0300067 (PMC10962806; doi:10.1371/journal.pone.0300067)
Supplement: S1 File — (DOCX) [file pone.0300067.s001.docx]

Inclusivity in global research

PLOS’ policy on inclusivity in global research aims to improve transparency in the reporting of research performed outside of researchers’ own country or community and ensures that PLOS publications reporting global research adhere to high standards for research ethics and authorship. Authors of relevant research articles may be asked to complete the questionnaire below, which outlines ethical, cultural, and scientific considerations specific to inclusivity in global research. This questionnaire may be requested when researchers have travelled to a different country to conduct research, if research uses samples collected in another country, research with Indigenous populations or their lands, or if research is on cultural artefacts. Researchers travelling to another country solely to use laboratory equipment will not normally be required to complete the questionnaire. However, the questionnaire can be requested at the journal’s discretion for any submission – if you have been requested to complete this questionnaire by the PLOS journal you submitted to, please do so.

Please complete the questionnaire below and include this as a Supporting Information file with your manuscript. Note that if your paper is accepted for publication, this checklist will be published with your article in the supporting information files. Please ensure that you reference the checklist in the main body of your manuscript. We suggest adding a subsection ‘Inclusivity in global research’ to your Methods section and adding the following sentence: “Additional information regarding the ethical, cultural, and scientific considerations specific to inclusivity in global research is included in the Supporting Information (SX Checklist)”

The questions have been designed to be applicable to a wide range of study types, and there are subsections for both human subjects research and non-human subjects research. If any of the questions are not relevant to your research please mark them as “N/A” as appropriate.

**Ethical considerations, permits and authorship**

*This section is applicable to all research types.*

Provide details as to who granted permissions and/or consent for the study to take place in the Methods section of your manuscript. This should include the names of **all** ethics boards, governmental organizations, community leaders or other bodies that provided approval for the study. If individuals provided approval refer to these people by their role or title but do not list their name(s).

Reported on page number: 11-12

If there were any deviations from the study protocol after approval was obtained please provide details of these changes in the Methods section of your manuscript.
Did this study involve local collaborators that are residents of the country where the research was conducted or members of the community studied? If you do not have any authors from said communities, please provide an explanation for this below.

Reported on page number: Unavailable

Our study was enriched by the collaboration with local partners Yanping Ying, Chuanyi Ning, Li Xue, and Xiao Pan, who are residents of the country where the research was conducted and members of the community studied. Each collaborator contributed significantly to different aspects of the research:

Yanping Ying played a pivotal role in the conceptualization, methodology, and supervision of the research project, ensuring that our approach was well-founded and effectively executed.

Chuanyi Ning was instrumental in writing, reviewing, and editing the manuscript, contributing to the clarity and quality of our research presentation.

Li Xue contributed to the methodology and visualization of our findings, in addition to writing, reviewing, and editing. Their expertise was crucial in accurately interpreting and presenting our data.

Xiao Pan took charge of preparing the original draft, reviewing, and editing the manuscript, and provided additional supervision, ensuring coherence and academic rigor.

Everyone listed as an author should meet PLOS’ criteria for authorship and all individuals who meet these criteria should be included in the author byline, rather than the acknowledgements. For further information please see the journal’s Authorship Policy.

**Human subjects research (e.g. health research, medical research, cross-cultural psychology)**

Did you obtain written informed consent from a representative of the local community or region before the research took place? How did you establish who speaks for the community? Details of written informed consent obtained from study participants should be reported separately in the Methods section of your manuscript.

Yes, prior to the initiation of our research, we obtained written informed consent from a representative of the local community or region.

The representative provided written informed consent after a comprehensive discussion about the research objectives, methods, potential risks, and benefits. This consent was documented in accordance with ethical guidelines and legal requirements, ensuring that the community's rights and welfare were fully protected.

Details of the written informed consent obtained from individual study participants, including how we informed them about the study's purpose, procedures, potential risks, and benefits, and their rights as participants.

How did members of the local community provide input on the aims of the research investigation, its methodology, and its anticipated outcome(s)?

In our study, we actively sought and incorporated feedback from local community members, including patients, healthcare professionals, and patient advocacy groups. This collaborative effort was facilitated through structured interviews, focus groups, and community meetings.

When engaging with the local community, how did you ensure that the informed consent documents and other materials could be understood by local stakeholders?

To ensure that members of the local community could understand the informed consent documents and other materials, we implemented the following measures:

Language and Cultural Sensitivity: All documents were translated into the local language(s) and culturally adapted.

Simplification of Materials: The language in the documents was simplified, reducing professional jargon and providing easy-to-understand explanations when necessary.

Pre-Test with a Sample Group: A pre-test was conducted with a sample group of local stakeholders to collect feedback and improve the clarity and understandability of the materials.

Use of Visual Aids: Visual aids such as diagrams, pictures, and flowcharts were included to help explain the study process and participants' rights.

Oral Explanations and Q&A Sessions: Oral explanations were provided during community meetings, with ample time for questions and answers to ensure participants' clear understanding of the study and their involvement.

Will the findings of the research be made available in an understandable format to stakeholders in the community where the study was conducted (e.g. via a presentation, summary report, copies of publications, etc.)? Please provide details of how this will be achieved.

Yes, the findings of the research will be disseminated to stakeholders in the community where the study was conducted. This will be achieved through various means such as presentations, summary reports, and accessible copies of publications. We understand the importance of making the information understandable and accessible to all stakeholders, and we are committed to ensuring effective communication channels to achieve this goal.

**Non-human subjects research using specimens/ animals collected as part of the study, or those housed in archival collections. Examples include archaeology, paleontology, botany and zoology.**

Did the permission you obtained from a local authority to perform the study include an agreement on access to outputs and benefit sharing? This may include procedures to enable fair distribution of the benefits and resources arising from the research performed. Please include any details of Prior Informed Consent and Benefit Sharing Agreements obtained. These may be required by field-specific regulations, for example the Convention on Biological Diversity (CBD) and the associated Nagoya Protocol.

If the material used in your study was imported, please A) provide the year it was imported and B) indicate whether permits were obtained to import/export the materials used, C) provide details of any permits obtained. If this information is not available, please indicate this.

If you used archival specimens, please state how the material used in your study was acquired by the institute it is held in and provide details of any permits obtained for the original excavations/ sample collection. If this information is not available, please indicate this.

How was the potential cultural significance of the materials collected in your study to local communities considered in your research design? Were Indigenous peoples and/or local researchers and institutions involved with archaeological excavations / collection of specimens? If so, please provide a description of their involvement.

If your manuscript includes photographs of human remains please indicate whether authors obtained permission from descendants or affiliated cultural communities to do so.
